# Happiness Detected by the Emotion Cognition System Is Associated with Burnout in an Information Technology Products and Services Trading Company

**DOI:** 10.3390/ijerph20032212

**Published:** 2023-01-26

**Authors:** Yasumasa Otsuka, Yukiko Sagisaka, Junko Nakamura, Keiko Hara, Masaki Okada, Yuko Takeuchi, Mizuki Tsuchiya, Yutaka Monden

**Affiliations:** 1 Faculty of Human Sciences, University of Tsukuba, 3-29-1 Otsuka, Bunkyo-ku, Tokyo 1120012, Japan; 2 R & D Center for Working Persons’ Psychological Support, University of Tsukuba, 3-29-1 Otsuka, Bunkyo-ku, Tokyo 1120012, Japan; 3 TEKWIND Co., Ltd., 3-19-11 Yushima, Bunkyo-ku, Tokyo 1130034, Japan

**Keywords:** burnout, emotion, happiness, emotion cognition system, worker

## Abstract

(1) Background: Although many previous studies have found an association between burnout and emotions, none have examined the association between emotions detected by an emotion cognition system and burnout. The purpose of this study is to examine the relationship between the emotions detected by the emotion cognition system and burnout among workers. We hypothesized that burnout survivors are less likely to express their emotions as facial expressions. (2) Methods: One hundred and forty-one workers at an Information Technology (IT) products and services trading company were asked to take facial images for three months when they started and left work and responded to a burnout questionnaire once a month. Microsoft Azure was used to detect their emotions. (3) Results: Hierarchical multiple regression analyses revealed that happiness in Period 1 was significantly and negatively associated with burnout at Time 2. This association was also observed after the various covariates were included. However, burnout at Time 3 was not significantly related to any emotions in Period 1. (4) Conclusions: Happiness, as detected by the emotion cognition system, was associated with burnout immediately afterward.

## 1. Introduction

Burnout, defined as “a syndrome conceptualized as resulting from chronic workplace stress that has not been successfully managed” [1], has been a major occupational health issue for many years. A systematic review conducted by Salvagioni et al. [2] revealed that burnout led to insomnia, depressive symptoms, fatigue, coronary heart disease, headaches, and other psychological and physical symptoms. Although burnout is not classified as a disease [1], it may lead to many psychological and physical symptoms or illnesses. Thus, it is important for employers and supervisors to manage their subordinates’ levels of burnout to protect their health.

Burnout has previously been studied in human service workers in terms of emotional labor. However, Leiter and Schaufeli [3] pointed out that human service workers’ burnout is only one form experienced in various occupations, and burnout research is now being conducted for a variety of workers. In fact, burnout studies have been conducted for forestry workers [4], electronics company workers [5], and other occupations.

Burnout consists of three components: emotional exhaustion, depersonalization, and reduced personal accomplishment [6]. Emotional exhaustion is feelings of energy depletion or exhaustion due to prolonged exposure to high job stressors. Depersonalization refers to the state in which one responds to others in an unkind and inhuman manner, having exhausted their emotional resources. Reduced personal accomplishment is the feeling of being less competent and less accomplished due to a sharp decline in performance. Among these three components, Maslach et al. [7] stated that “exhaustion is the central quality of burnout and the most obvious manifestation of this complex syndrome.” Thus, emotional exhaustion may be a better predictor of an employee’s burnout. Employees who feel burnout are exhausted both physically and psychologically, and are in a state of “emotional numbness” [8].

Positive emotions can broaden and build our thinking and behavior [9,10], which may lead to increased self-efficacy [11] and work engagement [12]. For example, Rogala [13] found that positive emotions increase job crafting, meaning ingenuity in one’s work [14], through a longitudinal study. Job crafting is strongly associated with self-efficacy [13] and work engagement [15]. Increasing job crafting by improving positive emotions may lead to a positive attitude toward work without giving up, resulting in an increased possibility of success at work and less emotional exhaustion.

Recently, Artificial Intelligence (AI) technology has advanced to detect our emotions from facial expressions. Several commercial emotion cognition systems have been developed, such as Amazon Rekognition, Baidu Research, Affectiva, and Microsoft Azure [16]. Most of the software can detect the type and percentage of each emotion in our facial expressions. Measuring emotions with questionnaires may introduce biases such as social desirability. However, if the emotions an individual is feeling can be detected from their facial expressions, burnout may be better detected.

The purpose of this study is to examine the relationship between the emotions detected by the emotion cognition system and burnout among workers. We hypothesized that burnout survivors, especially those who feel high levels of emotional exhaustion, feel wear and tear on their emotional resources due to chronic job stressors and are, therefore, less likely to express their emotions in their facial expressions.

## 2. Materials and Methods

### 2.1. Participants

The sample size was set to 82 calculated by G-Power 3.1.9.7 with *r* = 0.30, *α* = 0.05, and *β* = 0.80. The survey was conducted on 160 participants with an expectation of approximately 50% of the participants would drop out. A total of 141 employees (88 males and 53 females) from an IT products and services trading company participated in the study. After excluding 41 employees whose survey results were incomplete, 100 employees were included in the analyses (Table 1). The response rate was 88.1%. The mean age of the analyzed subjects was 38.0 years (*SD* = 9.8). Although the participants in our study were recruited from an IT products and services trading company, the actual occupations were in planning, purchasing, and sales, which are involved with others, accounting for 71.0% of the participants. Full-time employees accounted for 86.0% of the total.

### 2.2. Measures

#### 2.2.1. Burnout

The Japanese version of the Burnout Assessment Scale (BAT-J) [17] was used to assess burnout. The BAT-J consists of two aspects: core symptoms and secondary symptoms. The core symptoms consist of four subscales (exhaustion, mental distance, emotional impairment, and cognitive impairment) with 23 items, and the secondary symptoms consist of two subscales (psychological distress and psychosomatic complaints) with 10 items. In this study, the core symptoms and secondary symptoms were considered as one factor and were summed together (hereafter referred to as “BAT-J total”), following the recommendation of the Japan Ministry of Health, Labour and Welfare [18], as a criterion for measuring total levels of burnout. Responses were obtained using a 5-point Likert scale ranging from “never” (1) to “always” (5). No cutoff value for burnout existed for the BAT-J total [18].

#### 2.2.2. Emotions

Participants were asked to install the “Face Recognition and Attendance App” developed by TEKWIND Co., Ltd., including Microsoft Azure, on their smartphones and to take pictures of their faces when they started and left work on workdays. Before taking the pictures, participants were asked to select one time (starting work or leaving work) and one location (office, home, or other). However, the location at which the photos were taken was not included in this study because it was largely influenced by the department and the type of work. To capture natural facial expressions, facial images were taken twice: when the camera button was pressed and about 4 s after the button was pressed. The mean time interval between the first press and the second shot was 3.847 s. Since the first shot was likely to be expressionless because of the button press, we used the more natural facial expression a few seconds after the button was pressed. Eight emotion scores (anger, contempt, disgust, fear, happiness, neutral, sadness, and surprise) were obtained from the face image data captured from the second shot using Microsoft Azure’s estimations. The eight emotion scores estimated from a single facial image sum up to 1.0000. The study period was from 1 November 2021 to 7 February 2022.

#### 2.2.3. Other Variables

We also obtained information that was assumed to be related to burnout, such as personal characteristics, the number of days away from work due to illness (hereafter referred to as “absence days”), and major changes in work and personal life (hereafter referred to as “major changes”). Personal characteristics comprised gender, age, occupation, and employment type. The number of absence days was counted as 0.5 days for half-day leave, including paid leave, and responses were requested for each month from September 2021 to January 2022. Major changes included changes at work, changes in participants’ personal life, changes in their family life, and others. The first survey (Time 1) was conducted from 8 to 16 November 2021; the second (Time 2) from 15 to 24 December 2021; and the third (Time 3) from 8 to 16 February 2022.

### 2.3. Ethical Considerations

Prior to the survey, informed consent was obtained from all participants. Participants were informed that the survey would consist of the taking pictures of their own face and answering the questionnaire and that participation in the study was voluntary. Furthermore, participants were informed that they would not be disadvantaged in any way if they declined or withdrew their cooperation at any time. The Ethics Committee of the Faculty of Human Sciences, University of Tsukuba approved the study protocol (No. TOU 2021-34).

## 3. Results

### 3.1. Burnout, Absence Days, and Major Changes

Descriptive statistics of the BAT-J total, the number of absence days, and the major changes at each time are summarized in Table 2. The mean scores of the total BAT-J ranged from 81.3 (*SD* = 21.0) at Time 1 to 83.8 (*SD* = 22.1) at Time 3. For the number of absence days, “0 days” accounted for 76.0% in November and 86.0% in December and January. Participants who selected one or more of the four domains for major changes were defined as “Yes,” and the others were defined as “No.” The results showed that “Yes” was selected by 5.0% of the respondents at Time 2 and 8.0% at Time 3, indicating that a small number of participants experienced a major change in their work or private life during this period.

### 3.2. Facial Image and the Emotions

**Figure 1:**
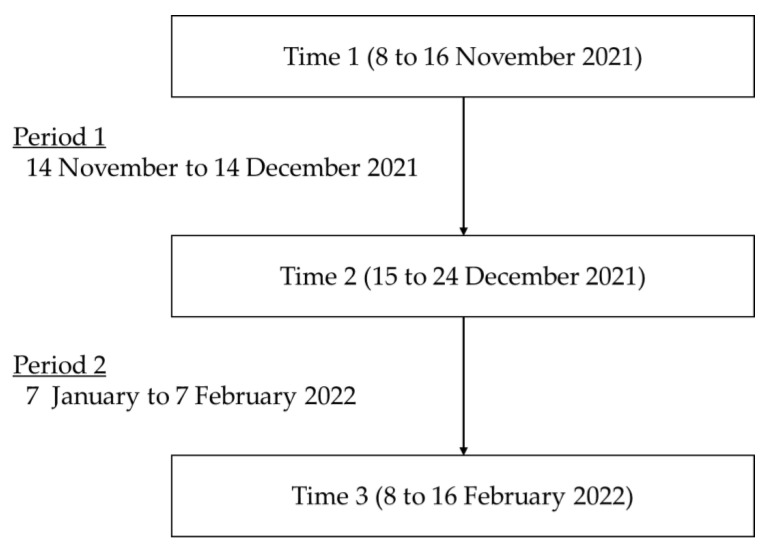
The timeline of the survey.

Two periods were set for the analysis. The first period (Period 1) was from 14 November to 14 December 2021, one month before Time 2, and the second period (Period 2) was from 7 January to 7 February 2022, one month before Time 3. The timeline of the survey is summarized in Figure 1. The mean score for the number of shots taken when starting work was 12.3 (*SD* = 6.3) for Period 1 and 10.9 (*SD* = 6.8) for Period 2, indicating that participants captured their face approximately half of the working days in a month. The mean score for the number of shots taken when leaving work was 10.3 (*SD* = 6.6) for Period 1 and 9.4 (*SD* = 7.1) for Period 2. Since the number of shots starting work was significantly higher than leaving work (Period 1: *t*(99) = 5.80, *p* < 0.001; Period 2: *t*(99) = 4.28, *p* < 0.001), the mean scores for emotions when starting work were used for the analyses.

The maximum and minimum mean scores for the eight emotions are shown in Table 3. The mean was calculated by averaging each emotional score at the start of each period for each subject. The maximum score for the neutral emotion was 0.9987 in Period 1 and 0.9997 in Period 2, and the minimum score was 0.4016 in Period 1 and 0.1670 in Period 2; this had the highest score among the eight emotions. The maximum score for happiness was 0.5824 in Period 1 and 0.5320 in Period 2; the maximum score for happiness was next to that of the neutral emotion. However, the maximum score for disgust was 0.0033 in Period 1 and 0.0069 in Period 2, and the maximum score for fear was 0.0089 in both Periods 1 and 2, all of which were lower than 0.01, and relatively low scores among the eight emotions. 

### 3.3. Association between Burnout and Emotions

To confirm the extent to which burnout is related to each emotion, the correlation between the BAT-J total scores at Time 1, Time 2, and Time 3 and the emotion scores when starting work in Period 1 and Period 2 were calculated by Pearson correlation coefficients (Table 4). The results showed that the BAT-J total of Time 2 had a significant negative correlation with happiness (*r* = −0.30, *p* < 0.01) and a significant positive correlation with surprise (*r* = 0.32, *p* < 0.01) and neutral emotion (*r* = 0.21, *p* < 0.05) in Period 1. The BAT-J total of Time 2 showed a significant negative correlation with happiness (*r* = −0.25, *p* < 0.05) and a significant positive correlation with the neutral emotion (*r* = 0.25, *p* < 0.05) in Period 2. The BAT-J total in Time 3 showed a significant negative correlation with happiness (*r* = −0.24, *p* < 0.05) and a significant positive correlation with surprise (*r* = 0.33, *p* < 0.01) in Period 1. The BAT-J total at Time 1 showed a significant negative correlation with happiness (*r* = −0.25, *p* < 0.05) and a significant positive correlation with surprise (*r* = 0.27, *p* < 0.01) in Period 1.

Next, we examined the relationship between the BAT-J total at Time 2 and the emotion scores in Period 1. To examine the degree to which emotion scores have explanatory power for burnout, we conducted a hierarchical multiple regression analysis (forced entry method) with the BAT-J total at Time 2 as the dependent variable; the emotion scores in Period 1 as the independent variable; and the BAT-J total at Time 1, number of shots in Period 1, personal characteristics, major changes at Time 2, and absence days as covariates (Table 5). Gender and major changes were scored as dummy variables. The neutral emotion was excluded from the analyses due to its high Variance Inflation Factor (VIF) score, implying multicollinearity with other variables.

The results showed that the explanatory rate for the BAT-J total of Time 2 with independent variables was 22%, which was significant at the 1% level (*F* = 3.713, *df* = 98). In Step 1, happiness in Period 1 had a significant negative effect (*β* = −0.27, *p* < 0.01), and surprise in Period 1 had a significant positive effect (*β* = 0.28, *p* < 0.01), on the BAT-J total at Time 2. In Step 2, happiness in Period 1 had a significant negative effect on the BAT-J total at Time 2, even after controlling for covariates (*β* = −0.09, *p* < 0.05).

To examine the long-term effects on burnout, a hierarchical multiple regression analysis was conducted using the BAT-J total of Time 3 as the dependent variable, the emotion scores of starting in Period 1 as the independent variables; and the BAT-J total at Time 1, number of shots in Period 1, personal characteristics, major changes at Time 3, and absence days as covariates (Table 6). The explanatory rate of the BAT-J total at Time 3 was 19%, which was significant at the 1% level (*F* = 3.131, *df* = 98). In Step 1, happiness in Period 1 had a significant negative effect (*β* = −0.22, *p* < 0.05), and surprise in Period 1 had a significant positive effect (*β* = 0.31, *p* < 0.01), on the BAT-J total at Time 2. However, in Step 2, when the covariates were entered, the emotion scores had no significant association with the BAT-J total at Time 3.

## 4. Discussion

This study examined the prospective relationships between emotions and burnout. One hundred and forty-one workers at an IT products and services trading company were asked to take facial images when they started and left work for three months and responded to a burnout questionnaire once a month. Correlation analysis revealed that the neutral emotion in Period 1 was significantly and positively correlated with burnout at Time 2, and the neutral emotion in Period 2 was also significantly and positively correlated with burnout at Time 2 and Time 3. Happiness in Period 1 was significantly and negatively correlated with burnout at all timepoints, and surprise in Period 1 was significantly and positively correlated with burnout at all timepoints. Hierarchical multiple regression analyses revealed that happiness in Period 1 was significantly and negatively associated with burnout at Time 2. This association was also observed after the various covariates were included. However, burnout at Time 3 was not significantly related to any emotions in Period 1. These results partially support the hypothesis that burnout survivors are less likely to express their emotions in facial expressions.

Hierarchical multiple regression analysis suggests that even after controlling for the effects of various covariates, the low expression of happiness may predict burnout immediately afterward. The mean score for happiness, as expressed over one month, ranged from 0.5824 to 0.0000, indicating that happiness is the most likely emotion to be expressed, except for the neutral emotion, by workers when starting work. Several previous studies showed a negative association between happiness and burnout. Sharif et al. [19] found a significant negative association between happiness and burnout in a study of 344 nurses working in hospitals in Iran. Similar associations were found in a study of 1147 European pilots [20] and a study of 548 German general practitioners [21]. Most of these studies were cross-sectional and administered questionnaires measuring burnout and happiness; thus, common method bias [22] may have occurred in these studies because they used the same measurement method. However, our study found the same association between emotions as detected by the face cognition system and burnout as measured by the questionnaire. Therefore, our results may corroborate the negative association between happiness and burnout. To the best of our knowledge, no previous study has reported a prospective association between happiness and burnout; our results may suggest that happiness, as detected by the face cognition system, may be an indicator predicting burnout immediately afterwards.

Although the results of the correlation analysis showed a positive association between neutral expression and burnout, it could not be entered as an independent variable in the hierarchical multiple regression analysis since multicollinearity was observed for the neutral emotion. While Microsoft Azure is considered to be able to recognize emotions relatively more accurately than other face cognition systems [16,23], it is recognized that it also detects many neutral emotions [16]. Since the maximum detection range for neutral emotions in this study was extremely high, multicollinearity was more likely to be observed when other emotions were included as independent variables. Thus, it is necessary to reexamine the relationship with burnout using other face cognition systems in the future.

Several limitations of this study should be noted. First, the survey was conducted on employees working for one Japanese IT products and services trading company, but 71.0% of the participants had roles other than IT engineers, such as planning, purchasing, and sales. Therefore, there is a possibility that these results can be applied to other organizations, although further study should be warranted. Second, the frequency at which facial images were taken varied between participants. In this study, since we did not force participants to take pictures, the number of shots that were taken could not be kept constant. Although the average score for each emotion for each participant over one month was used as an indicator, it will be necessary to conduct continuous filming to examine the relationship between changes in emotions and burnout, since the expression of emotions changes from day to day. Third, data are missing for those who did not participate in the study. Those who had burned out during or before participation were not included in this study, and this study may not have shown the real association between emotions and burnout.

Finally, we offer some practical implications from this study. Since burnout may occur within one month if the expression of happiness is low, supervisors should consider this as a state in which burnout risk is increasing in their subordinates. Active monitoring and early detection of signs of burnout, and expressing less happiness, may give more opportunities for supervisors to take care of their subordinates.

## 5. Conclusions

We examined the association between emotions obtained from facial images, captured by the face cognition system, and subsequent burnout. Results revealed that happiness in Period 1 was significantly and negatively associated with burnout at Time 2. This association was also observed after the various covariates were included. However, burnout at Time 3 was not significantly related to any emotions in Period 1. These results indicate that burnout may occur within one month if the expression of happiness is low among employees in an IT products and services trading company.

## Figures and Tables

**Table 1:** Study participants (*n* = 100).

		*n*	%
Gender	Male	64	64.0
	Female	36	36.0
Age	20–29	24	24.0
	30–39	30	30.0
	40–49	31	31.0
	50–59	13	13.0
	60-	2	2.0
Occupation	Planning	27	27.0
	Purchasing	14	14.0
	Sales	30	30.0
	Manufacturing	10	10.0
	Logistics	19	19.0
Employment Type	Full-time	86	86.0
	Contract	2	2.0
	Part-time	12	12.0

**Table 2:** Descriptive statistics of burnout, absence days, and major changes.

		Time 1		Time 2		Time 3	
	Range	Mean	SD	Mean	SD	Mean	SD
Burnout							
Total	33–165	81.3	21.0	82.9	22.2	83.8	22.1
Core symptoms	23–115	56.8	14.5	58.1	15.4	58.6	15.5
Exhaustion	8–40	23.0	5.8	23.3	6.0	23.3	6.2
Mental distance	5–25	10.8	3.9	11.5	4.1	11.5	4.1
Emotional impairment	5–25	10.8	3.6	11.0	3.8	11.4	3.5
Cognitive impairment	5–25	12.2	3.7	12.4	4.0	12.5	4.0
Secondary symptoms	10–50	24.5	7.5	24.7	7.9	25.1	7.7
Psychological distress	5–25	12.7	4.2	12.8	4.3	13.0	4.0
Psychosomatic complaints	5–25	11.8	3.8	12.0	4.2	12.2	4.2
		Time 1		Time 2		Time 3	
		*%*		*%*		*%*	
Absence (days)							
September	0	84.0					
	0.5–1.5	13.0					
	2 or more	3.0					
October	0	83.0					
	0.5–1.5	13.0					
	2 or more	4.0					
November	0			76.0			
	0.5–1.5			16.0			
	2 or more			8.0			
December	0					86.0	
	0.5–1.5					8.0	
	2 or more					6.0	
January	0					86.0	
	0.5–1.5					13.0	
	2 or more					1.0	
Major changes							
	Yes			5.0		8.0	
	No			95.0		92.0	

**Table 3:** Maximum and minimum mean of the emotion scores.

	Period 1		Period 2	
Emotion	Max	Min	Max	Min
anger	0.0143	0.0000	0.0360	0.0000
contempt	0.0771	0.0000	0.2930	0.0000
disgust	0.0033	0.0000	0.0069	0.0000
fear	0.0089	0.0000	0.0089	0.0000
happiness	0.5824	0.0000	0.5320	0.0000
neutral	0.9987	0.4016	0.9997	0.1670
sadness	0.2108	0.0000	0.3683	0.0002
surprise	0.3100	0.0000	0.0761	0.0000

**Table 4:** Pearson correlation coefficients between burnout and emotions.

				Burnout			
Period	Emotion	Time 1		Time 2		Time 3	
Period 1	anger	0.09		0.15		0.08	
	contempt	−0.01		−0.09		−0.01	
	disgust	0.09		0.11		0.13	
	fear	0.05		0.09		0.06	
	happiness	−0.25	*	−0.30	**	−0.24	*
	neutral	0.16		0.21	*	0.15	
	sadness	−0.09		−0.11		−0.11	
	surprise	0.27	**	0.32	**	0.33	**
Period 2	anger	−0.08		−0.08		−0.04	
	contempt	−0.16		−0.19		−0.16	
	disgust	−0.12		−0.12		−0.07	
	fear	−0.02		0.03		0.05	
	happiness	−0.19		−0.25	*	−0.20	
	neutral	0.20		0.25	*	0.23	*
	sadness	−0.04		−0.04		−0.09	
	surprise	−0.05		0.01		0.03	

** *p* < 0.01, * *p* < 0.05.

**Table 5:** Results of hierarchical multiple regression analysis for burnout at Time 2.

		Step 1			Step 2		
		*β*	*t*		*β*	*t*	
Emotions in Period 1	anger	0.12	1.18		0.05	1.14	
	contempt	−0.07	−0.59		−0.06	−1.04	
	disgust	0.13	1.22		0.06	1.18	
	fear	0.02	0.20		0.02	0.43	
	happiness	−0.27	−2.87	**	−0.09	−2.00	*
	sadness	−0.13	−1.28		−0.03	−0.54	
	surprise	0.28	2.98	**	0.06	1.31	
Number of shots in Period 1					−0.06	−1.32	
Burnout	BAT-J total at Time 1				0.82	17.28	***
Personal characteristics	Gender				0.00	−0.08	
	Age				0.01	0.30	
Absence days	September				0.06	1.15	
	October				−0.05	−0.91	
	November				0.06	1.01	
Major changes	Major changes at Time 2				0.07	1.51	
	*R²*		0.22	**		0.85	***
	*ΔR²*					0.63	***

*** *p* < 0.001, ** *p* < 0.01, * *p* < 0.05.

**Table 6:** Results of hierarchical multiple regression analysis for burnout at Time 3.

		Step 1			Step 2		
		*β*	*t*		*β*	*t*	
Emotions in Period 1	anger	0.06	0.60		−0.03	−0.61	
	contempt	0.00	0.03		0.09	1.41	
	disgust	0.14	1.28		0.02	0.26	
	fear	−0.01	−0.08		−0.01	−0.29	
	happiness	−0.22	−2.29	*	0.02	0.44	
	sadness	−0.13	−1.27		−0.02	−0.36	
	surprise	0.31	3.25	**	0.05	0.93	
Number of shots in Period 1					0.01	0.11	
Burnout	BAT-J total at Time 1				0.89	16.52	***
Personal characteristics	Gender				−0.02	−0.46	
	Age				0.00	−0.08	
Absence days	October				−0.04	−0.70	
	November				0.07	0.96	
	December				−0.03	−0.42	
Major changes	Major changes at Time 3				0.07	1.51	
	*R²*		0.19	**		0.82	***
	*ΔR²*					0.63	***

*** *p* < 0.001, ** *p* < 0.01, * *p* <0.05.

## Data Availability

Not applicable.
